# Supplementary material for: Lifetime homelessness among young transgender women in Lima, Peru is associated with HIV vulnerability: Results from a cross-sectional survey
Source: PLOS Glob Public Health. 2025 Apr 21;5(4):e0004351. doi: 10.1371/journal.pgph.0004351 (PMC12011236; doi:10.1371/journal.pgph.0004351)
Supplement: S1 Table — (DOCX) [file pgph.0004351.s001.docx]

# **S1 Table: HIV and STI vulnerability among young transgender women with and without experiences of homelessness (categorical homelessness)**

|  | **Homeless in past 3 months**  **N = 19**  n (%) | **Homeless >3 months ago**  **N = 49**  n (%) | **Never homeless**  **N = 141**  n (%) | **P-value** |
| --- | --- | --- | --- | --- |
| **HIV status^1^** |  |  |  | 0.06 |
| Positive | 11 (68.8) | 14 (35.0) | 42 (39.6) |  |
| Negative | 5 (31.2) | 26 (65.0) | 64 (60.4) |  |
| **Bacterial STI^2^** |  |  |  | 0.33 |
| Positive | 7 (46.7) | 10 (25.6) | 32 (31.1) |  |
| Negative | 8 (53.3) | 29 (74.4) | 71 (68.9) |  |

^1^ Among n=162 participants who completed HIV testing

^2^ Among n=157 participants with complete tests for syphilis (TPHA positive, RPR > 1:8), gonorrhea, (pharyngeal) or chlamydia (pharyngeal)

Tests for significance estimated with missing data excluded using global chi-square test; Fisher’s exact test for variables with cell ≤5
