# Supplementary material for: Lifetime homelessness among young transgender women in Lima, Peru is associated with HIV vulnerability: Results from a cross-sectional survey
Source: PLOS Glob Public Health. 2025 Apr 21;5(4):e0004351. doi: 10.1371/journal.pgph.0004351 (PMC12011236; doi:10.1371/journal.pgph.0004351)
Supplement: S2 Table — (DOCX) [file pgph.0004351.s002.docx]

**S2 Table: Poisson regression models with robust standard errors estimating the association between recent homelessness and past 6-month condomless sex with a partner living with HIV or status unknown partner among young transgender women in Lima, Peru**

|  | **Model 1**  PR (95% CI) | **Model 2**  aPR (95% CI) | **Model 3**  aPR (95% CI) |
| --- | --- | --- | --- |
| **Homelessness**  Never (ref)  Recent (past 3 months)  Lifetime (more than 3 months ago) | 1.00 (ref)  1.30 (0.71-2.39)  2.11 (1.55-2.89) | 1.00 (ref)  1.32 (0.72-2.42)  2.15 (1.54-3.00) | 1.00 (ref)  1.18 (0.69-2.00)  1.54 (1.10-2.15) |
| **Age** (per 1 year increase) | -- | 0.98 (0.91-1.06) | 0.96 (0.89-1.04) |
| **Secondary school completion** (Ref = no secondary school) | -- | 0.85 (0.60-1.21) | 1.08 (0.77-1.50) |
| **Sex work** (Ref = never) | -- | -- | 3.75 (1.93-7.31) |
| **Non-injection drug use** (Ref = no use) | -- | -- | 1.13 (0.79-1.60) |

All models included N=176 participants: N=180 had complete data on the outcome (condomless sex in the past 6 months) and N=4 were excluded due to missing covariate data).

Abbr: PR: Prevalence ratio; aPR: adjusted prevalence ratio; 95% CI: 95% confidence interval.
