# Supplementary material for: Lifetime homelessness among young transgender women in Lima, Peru is associated with HIV vulnerability: Results from a cross-sectional survey
Source: PLOS Glob Public Health. 2025 Apr 21;5(4):e0004351. doi: 10.1371/journal.pgph.0004351 (PMC12011236; doi:10.1371/journal.pgph.0004351)
Supplement: Attachment — Submitted filename: PLOS_Peru-YTW_Housing_Response_RR1.docx [file pgph.0004351.s005.docx]

Thank you for the opportunity to submit a revised draft of our manuscript titled “Lifetime homelessness among young transgender women in Lima, Peru is associated with HIV vulnerability: Results from a cross-sectional survey.”

We appreciate the time and effort that you and the reviewers have dedicated to providing valuable and thoughtful feedback on our manuscript. Below is a detailed response letter indicating changes made based on the reviewers’ feedback. Original reviewer comments are in bold.

**Reviewer #1**

Introduction:
**1. In reading the introduction, I felt that the justification for focusing on transgender women in this analysis was clear (few estimates of homelessness among transgender women, key population for HIV, often experience violence and other structural exposures that influence HIV risks). It was not immediately apparent to me why the authors chose to restrict their study to only young transgender women. Can the authors provide further information on to why this study population in particular was chosen?**

Thank you for this comment. To further clarify why this study focussed on young transgender women in particular, the following sentence have been added to the last paragraph of the introduction: ***“Further, studies conducted in Latin America have found that transgender women aged 18-24 are at a higher risk of HIV incidence and less likely to engage in HIV prevention strategies such as condom use and PrEP (28,29).”*** (Page 4, lines 69-71).

**2. Potential typo on page 3, line 26 with “ages 15-24”. Elsewhere in the manuscript, including in the methods, the young study population is defined using ages 16-24.**

Thank you, this has been fixed.

**3. In the introduction, a lot of information is given about how homelessness may influence HIV vulnerabilities through restricting access to healthcare services (e.g., HIV treatment uptake and adherence, PrEP adherence, discrimination for healthcare workers, lack of health insurance etc.) and these pathways appear to be the focus, but these were not investigated in the regression analysis, which focused on links with condom use. Could the authors provide further justification/hypotheses that explain why they chose to focus on the analysis variables of interest? For example, in the introduction there is no mention of PTSD or non-injection drug use, which are important variables in the regression models.**

Thank you for this suggestion. The second and third paragraph of the introduction (pages 3-4) has been re-worked to clarify the focus on condomless sex as a key risk factor for HIV acquisition in this population. Further details have been added to explain how poor mental health, substance use, and violence can decrease condom use as well as increase the risk of homelessness (new text in bold):

*“Homelessness can also decrease the ability to negotiate condom use with paying and non-paying sexual partners in the context of economic precarity (e.g., being offered more money to engage in condomless sex), particularly for individuals who are relying on friends, intimate partners, or sex work clients for temporary housing (19,20).* ***Condomless sex is a key pathway for HIV acquisition and transmission among transgender women (24). Poor mental health, substance use, and interpersonal violence can all contribute to decreased condom use as well as increased homelessness [25,26].”***

**Methods:**
**4. Page 5, line 77. Perhaps more information (or at least a reference, if available) on the transfeminine continuum would be useful for readers who are less familiar? I notice this information is also not provided in your previous publication on this study.**

For this study, the language of “transfeminine continuum” was used to be inclusive of feminine gender identities among individuals assigned a male sex a birth, including identifying as a trans woman, as a woman, or other feminine non-binary identities. We have added a reference to an article explaining the use of the term to the study eligibility section (page 5, line 86).

**5. Page 5, lines 79-80: “Participants were recruited by peers and surveys were administered by peer survey interviewers”. From your previous paper it appears that snowball sampling was used? If so, I would specify this.**

Thank you for pointing this out. The community-based recruitment methods and the need to recruit individuals from this specific population meant that in addition to regular community-based recruitment methods, participants were encouraged to spread information about the study within their social networks. The “study design and participants” section has been revised to include the following (new text in bold):

*“Participants were recruited by peers* ***and recruiters from a community organization*** *and surveys were administered by peer survey interviewers.* ***Study participants were also invited to encourage potential participants from their social networks to contact the study team.”*** (page 5, lines 88-90).

**6. Page 5: Measures. In the study, questions were asked about lifetime homelessness, past 3 months homelessness, and past 30 days homelessness, as well as people’s current living situation. I would like some further information as to why the authors chose to focus on lifetime exposure as their primary exposure measure. In the discussion, it is stated that this is due to limited sample size, which presumably means too few people responded yes to questions about homelessness over shorter time periods than lifetime. Fine. My concern, however, is that the interpretation of the estimates generated with these analyses is muddled, because for some participants lifetime exposure could have been yesterday, while for others it could have been ~10 years ago or so. As such, the findings of these analyses do not seem to be particularly informative or actionable, and estimates based on more recent exposure may perhaps be more useful.**

Based on the limited pre-existing research on this topic as well as the young age of the study population, we chose to focus on lifetime homelessness as an exploratory question in this study. We have noted the exploratory nature of this question in the introduction (page 5, line 76). The purpose of these results is to demonstrate that a single episode of homelessness, particularly in a population this young, can be associated with increased HIV risk. This underscores both the urgency of preventing homelessness in this population as well as the importance of considering history of homelessness in clinical settings (e.g., screening for homelessness as a social determinant of HIV).

Your comment prompted interesting and important discussions on our team regarding how to operationalize “recent” homelessness within a population with such a high rate of lifetime homelessness. One option is a binary exposure (homeless in the past 3 months vs not homeless in the past 3 months), which leaves a substantial number of people with lifetime but not recent homelessness in the “unexposed” category. Another option is to consider a 3-level exposure variable (recent homelessness/lifetime homelessness/never homeless). We had conducted this analysis as a sensitivity analysis and have now included it in the manuscript, as well as a figure presenting the prevalence of condomless sex among participants with recent homelessness, lifetime homelessness, and no experiences of homelessness. However, due to the small number of participants experiencing recent homelessness, the estimates were less precise, particularly when adding covariates to the model.

The following text has been added to the manuscript to describe the new figure:

***“Participants with a history of homelessness were more likely to report condomless sex with a partner living with HIV or status unknown partner in the past 6 months (51.5%) compared to those who had never been homeless (29.1%). Among participants with complete data on condomless sex, 43.8% of participants who had been homelessness in the past 3 months reported condomless sex in the past 6 months (Figure 1).”*** (page 11, lines 216-220)

In addition, the methods and results of the sensitivity analysis have been added to the methods and results sections:

***“A sensitivity analysis was conducted where homelessness was operationalized as a categorical variable (homeless in the past 3 months, lifetime homelessness, never homelessness).”*** (page 8, lines 175-177)

***“When conducting the sensitivity analysis with homelessness operationalized as a 3-level variable (recent homelessness, lifetime homelessness, never homeless), the estimates for lifetime homelessness compared to never experiencing homelessness were similar (Supp Table 2). Smaller effect sizes with wider confidence intervals were observed for the association between recent homelessness and recent condomless sex.”*** (page 18, 297-302)

Finally, due to the cross-sectional and exploratory nature of this study and this secondary analysis in particular, the recall periods of several variables meant that we felt as though the lifetime homelessness exposure provided the most useful results. In particular, condomless sex was measured over the past 6 months while “recent’ homelessness had a recall period of 3 months. We are hopeful that this exploratory analysis will prompt further research, particularly longitudinal research, to better explore some of these aspects.

**7. Page 7: Statistical analysis. Throughout, there is a lot of focus on significance testing using p<0.05 to compare differences in characteristics between the two homelessness groups. The approach of relying only on p-values is known to be misleading (See:** [**https://pmc.ncbi.nlm.nih.gov/articles/PMC1119478/**](https://pmc.ncbi.nlm.nih.gov/articles/PMC1119478/)**), therefore I would put let emphasis on those.**

Thank you for this comment. The results section has been revised to reduce the focus on significant testing.

**8. Complete case analysis: For the regression models, why did the authors decide the do a complete case analysis? (i.e., exclude missing data on the covariates) Did the authors attempt to account for missing data in their regression models, e.g., using the missing indicator method? (**[**https://arxiv.org/abs/2111.00138**](https://arxiv.org/abs/2111.00138)**) Additionally, does the analytical sample of 176 apply to all three regression models?**

Yes, the analytical sample of 176 applies to all three regression models. The footnote on Table 5 has been edited to clarify this. After excluding participants with missing data on the exposure or outcome, 1 participant was missing age data while 3 were missing non-injection drug use.

Due to the exploratory nature of this study as well as the relatively small amount of missing covariate data (~2% of the analytical sample), we did not implement any imputation strategies.

**9. Regression approach: I am a bit confused about the regression approach. Can the authors explain why they chose to run three models, and sequentially incorporate different potential confounders in each? How were the variables to add in each model selected? Was selection based on statistical criteria (e.g., in stepwise selection) or based on prior causal hypotheses of these links?**

The models were constructed based on prior causal hypotheses, particularly regarding risk factors for the outcome, condomless sex. The variables were added sequentially using a block-wise modeling approach in order to examine different aspects of risk (e.g., behavioural, psychosocial). By sequentially entering blocks of variables conceptually grouped, we were able to assess the effect estimates in a block-wise fashion to understand their association with the outcome of condomless sex.

The following text has been revised in the methods section to provide more details on the modelling strategy in the statistical analysis section (new text in bold):

*“Poisson regression models with robust standard errors were fit to estimate the association between lifetime homelessness and past 6-month condomless sex in unadjusted and adjusted models with* ***covariates added in blocks*** *(48,****49****). In the first model, we adjusted for sociodemographic characteristics (age and education). In the second model, we adjusted for sociodemographic characteristics and* ***key*** *behavioural* ***risks that can increase engagement in condomless sex*** *(sex work and non-injection drug use). In the third model, we adjusted for sociodemographic characteristics, behavioural risks, and psychosocial* ***vulnerabilities that can increase engagement in condomless sex as well as homelessness*** *(PTSD and violence).* (page 8, lines 167-174).

A sensitivity analysis was conducted using psychological distress rather than PTSD and the results were unchanged.

**10. Furthermore, I am not sure about all the variables included in the models. In particular, I am not sure about adjusting for sex work and non-injection drug use as potential confounders of the association between homelessness and condom use. Rather, I see these variables as potential mediators (and can similarly view violence and PTSD as potential mediators). Adjusting for potential mediating variables could induce collider-stratification bias and result in spurious associations if there are unmeasured confounders of the mediator and outcome (**[**https://onlinelibrary.wiley.com/doi/10.1111/ppe.12474**](https://onlinelibrary.wiley.com/doi/10.1111/ppe.12474)**). Additionally, there may be the possibility that by adjusting for these variables you are blocking the pathways by which homelessness may influence the outcome, which results in the “non-significant” association in model 4. As such, I find the results of models 1 and 2 more useful than models 3 and 4. Therefore, can the authors provide further explanation for why they chose to adjust for these behavioural and psychosocial variables as confounders?**

Thank you for this comment, which highlights many of the difficulties in constructing models with homelessness-related exposure variables and HIV vulnerability outcomes due to the highly complex causal mechanisms, particularly given the cross-sectional nature of these data. While we agree with the concerns regarding the timing of the variables, we were limited by the structure of the data. As this is a secondary analysis of cross-sectional data, it is possible that some of these covariates are on the causal pathway. This was a factor in our decision to also present the unadjusted model and the model with only sociodemographic characteristics. However, we believed that the importance of behavioural and psychosocial characteristics as potential confounders, particularly for their potential associations with condomless sex, merit their inclusion in the models. These models (models 3 and 4) are particularly important to inform broader discussions surrounding HIV prevention interventions for young transgender women in Latin America and to prompt further research. The new language added to the introduction (page 4, lines 56-58) and methods (page 8, lines 169-173) sections also aims to explain the use of these variables.

**Results:

11. The authors found that 67 participants tested positive for HIV. Is there any indication of how many of these participants knew they were living with HIV before the study started?**

Among these 67 participants, 42% reported the result of their last HIV test was negative, 21% reported they were living with HIV, and the remaining 37% declined to answer the question regarding HIV status or reported it as unknown. Due to the high proportion of missingness/unknown results, we were not confident in providing estimates regarding how many cases of HIV could be considered “new.”

**12. I find that the descriptive results in this manuscript are particularly interesting, and I like the authors focus on these findings in the results section. It is an awful lot of information however, so it would be nice, if possible, to see some of it in a figure or two rather than only in tables.**

Thank for this comment regarding the descriptive results. Due to the variation in the types of variables included in the tables (continuous, binary, categorical) as well as wanting to be clear about the varying amount of data missingness, we felt that tables continued to be the clearest way to present these results. However, we will be attentive to this in future work.

**13. As stated in a previous comment, I would focus less on p-values for comparing differences, and just report those that seem most interesting. Even if the p-value is <0.0001 this does not necessarily suggest a difference between the groups and could just be due to chance, particularly as you are comparing lots of groups.**

Thank you for this comment. We have reduced the emphasis on statistical significance in the results section to focus more on presenting the descriptive results.

**14. Page 14, lines 241-243. I think the word “without” is missing from this sentence.**

Thank you, this has been fixed.

**Discussion:

15. My main concern in the discussion is the use of strong causal language to describe what were only observational cross-sectional associations in your study. On page 18, the paragraph beginning with “Consistent with research in other contexts…” the authors state that “we found that lifetime homelessness increased behavioural and structural vulnerability to HIV through increased likelihood of recent condomless anal sex, increased engagement in sex work, and increased likelihood of experiencing violence and poor mental health”. However, the analysis did not show this, it demonstrated cross-sectional links between homelessness and condom use, when adjusting for different variables, and the associations were not consistent in the 4 models run. As this was a cross-sectional analysis, we are not able to infer causality or exclude the possibility of reverse causality in relationships. I think the authors need to be more attentive to this in their discussion and in places soften the language to highlight the observed links without implying a causal or temporal direction e.g., “those who ever experienced homelessness were more likely to have engaged in sex work” rather than “homelessness increased engagement in sex work” etc. The authors acknowledge in the limitations that the design of the study limits causal inference, but the language used through the discussion implies they think the findings of the analyses represent causal relationships.**

Thank you for this comment. We have revised the specific sentence to read (new text in **bold**):

*“Consistent with research in other contexts, we found that lifetime homelessness* ***was associated with*** *behavioural and structural vulnerability to HIV,* ***with participants who had experienced homelessness more likely to report*** *recent condomless anal sex, engagement in sex work (both lifetime and in the past 30 days), and experiences of violence and poor mental health (22,47,57).”* (page 20, lines 353-357).

We have also carefully reviewed the results and discussion section, as well as the abstract, with attention to removing causal language and ensuring associational language only.

**Reviewer #2:**

**Introduction:
1. It would help to better anchor your intro in terms of the extent to which you are starting with global data then zooming in on peru.**

Thank you for this suggestion. Given the limited available global literature on homelessness among young transgender people we are unable to include more global literature. However, we have edited the first paragraph to better clarify that the available literature is coming from North America including the US and Canada. Further, the majority of research on homelessness and HIV has been conducted in high-income countries, with some exceptions (see for example Arum et al, 2021). We hope this paper can further justify the need to conduct more research on homelessness and housing insecurity in other global contexts, such as Peru.

**Methods:
1. Employment status: to what extent does that consider sex work as work?**

Participants were asked if they were currently working, with response options including a full time or part time job with a salary, informal work, or not working. We did not ask the nature of that work or provide any prompts about whether or not to consider sex work as work and sex work was probed in a separate section of the survey. Interestingly, 76% of participants who reported sex work in the past 30 days reported that they had “informal” employment, though we do not know for certain if sex work was the only form of informal employment they had. To further clarify, we have added the following sentence to the methods:

***“Employment status was assessed as formal employment (full time or part-time job with a salary), informal work, or unemployment.”*** (page 6, lines 114-116).

**2. Please clarify to what extent have these scales been validated in Spanish?**

The Transgender Community Connectedness Scale, the Interpersonal Sexual Objectification Scale, and the AUDIT-C scale have all been validated in Spanish while the transgender-specific intimate partner violence scale as well as the primary care PTSD Screen have not. The Kessler 6-item psychological distress scale has mixed validity in Spanish among Hispanic/Latino populations in the United States (see Kim et al, 2017) but to our knowledge has not been validated in Spanish in Latin America. A citation to the TCC Spanish validation study has been added to the methods section as well as clarifying language regarding the use of the Spanish-language ISOS and AUDIT-C scales.

We have also estimated Cronbach’s alpha for each of these scales to assess their reliability within our sample. All scales displayed good reliability, with alpha > 0.8, with the exceptions of the AUDIT-C scale (alpha = 0.69) and the Transgender Community Connectedness Scale (alpha = 0.4). Based on these results, we have removed the Transgender Community Connectedness Scale from the results.

We have incorporated these results into the manuscript in the results section:

***“The trans-specific intimate partner violence scale (α=0.94), interpersonal sexual objectification scale (α=0.95), primary care PTSD screen for DSM-V (α=0.93) and Kessler 6-item psychological distress scale (α=0.88) displayed good to excellent reliability while the AUDIT-C scale displayed lower reliability (α=0.69).”*** (page 14, lines 251-255)

In the discussion, we have also added the following sentence:

***“In addition, some scales (transgender-specific intimate partner violence, PC-PTSD) have not yet been validated in Spanish.”*** (page 22, lines 393-395).

**3. Please provide v brief context on Peruvian health system to better locate the extent to which health insurance is universal, employment specific etc - in general read through with this lens that readers may not have in depth knowledge of the country specific health system so consider spelling out / providing definintions and context even if only v briefly.**

Thank you for this comment. The following sentence has been added to the methods section to clarify the health insurance options:

***“Health insurance options included public insurance available to individuals living in poverty; private insurance paid for by the participant, their employer, or their family member; or no health insurance.”*** (page 7, lines 137-139).

We have carefully reviewed the manuscript, particularly the discussion, with attention to adding context where possible and ensuring clear and consistent language.
